# Macromolecular crowding explains overflow metabolism in cells

**DOI:** 10.1038/srep31007

**Published:** 2016-08-03

**Authors:** Alexei Vazquez, Zoltán N. Oltvai

**Affiliations:** 1 Beatson Institute for Cancer Research, Glasgow, UK; 2 Departments of Pathology and Computational & Systems Biology, University of Pittsburgh School of Medicine, Pittsburgh, PA, USA

## Abstract

Overflow metabolism is a metabolic phenotype of cells characterized by mixed oxidative phosphorylation (OxPhos) and fermentative glycolysis in the presence of oxygen. Recently, it was proposed that a combination of a protein allocation constraint and a higher proteome fraction cost of energy generation by OxPhos relative to fermentation form the basis of overflow metabolism in the bacterium, *Escherichia coli*. However, we argue that the existence of a maximum or optimal macromolecular density is another essential requirement. Here we re-evaluate our previous theory of overflow metabolism based on molecular crowding following the proteomic fractions formulation. We show that molecular crowding is a key factor in explaining the switch from OxPhos to overflow metabolism.

Life, at its most basic level, is a series of biochemical processes executed and constrained by the physicochemical properties and makeup of the cell. One key feature is that metabolic processes occur within the highly crowded, gel-like interior of the cell and its organelles^1^,^2^. Using a flux balance model (FBA) of *E. coli* metabolism and average estimates of enzyme kinetic parameters, we have shown that incorporating a molecular crowding constraint into FBA results in theoretical predictions that agree closely with experimental observations. Molecular crowding explains four phenomena: 1- hierarchy of substrate utilization by *E. coli* cells growing in a mix of carbon sources^3^,^4^, 2- the maximum growth rate of *E. coli* on different carbon sources and 3- their reduction upon genetic perturbations^3^, and 4- the excretion of acetate during fast growth^5^.

More recently, Adadi *et al*. have demonstrated that more precise estimations of enzyme kinetic parameters significantly improves the agreement between model predictions and experimental measurements in *E. coli*^6^. Similar mathematical results were obtained in the context of overflow metabolism in proliferating mammalian cells (Warburg effect^7^,^8^) and non-dividing muscle cells (lactate switch^9^), and in bacteria that do not display overflow metabolism or OxPhos but undergo growth-rate-dependent metabolic switches^10^.

A recent theoretical analysis of Basan *et al*.^11^ challenges the molecular crowding hypothesis of overflow metabolism by claiming that it simply originates from a protein allocation phenomenon. However, as we show here, their formulation contains implicit assumptions that expand beyond the hypothesis of protein allocation alone. More importantly, these additional assumptions are consistent with the molecular crowding hypothesis of overflow metabolism.

## Results

Following Basan *et al*.^11^, we divide the proteome into four fractions


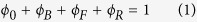


that are associated with proteins in non-metabolic pathways, ribosomes, fermentation and OxPhos, respectively. We further assume that the cell growth is in a steady state where the production and consumption of proteins, energy and carbon atoms is balanced


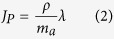



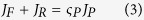



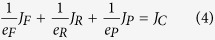


where *λ* is the growth rate, *J*_*P*_ is the rate of protein synthesis (moles of amino acids per cell volume and time), *J*_*F*_ is the rate of fermentation (in units of moles of ATP produced per unit of cell volume and time), *J*_*R*_ is the rate of OxPhos (in units of moles of ATP produced per unit of cell volume and time), *J*_*C*_ is the rate of carbon uptake (in units of carbon atoms per unit of cell volume and time), *ρ* is the protein density (mass of protein per cell volume), *m*_*a*_ is the average molecular weight of amino acids in expressed proteins, *ζ*_*P*_ is the energy consumption rate per unit of amino acid added to proteins, and *e*_*i*_ are the yields per unit of carbon atom. We consider the following kinetic models relating biochemical rates to enzyme concentrations.


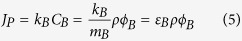



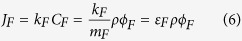



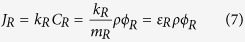


where *k*_*i*_ are effective turnover rates per unit of enzyme, *C*_*i*_ are enzyme concentrations, *m*_*i*_ are enzyme molar masses, and *ε*_*i*_ are effective turnover rates per unit of enzyme proteome fraction. By eliminating *J*_*p*_, *ϕ*_*B*_, *ϕ*_*F*_ and *ϕ*_*R*_ from equations (1–7) we obtain.


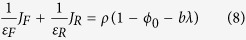



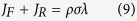



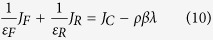


where


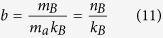



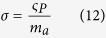



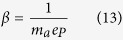


and *n*_*B*_ is the number of amino acids per ribosome. Except for the pre-factor *ρ* in equations (8–10), these equations are equivalent to the equations derived by Basan *et al*.^11^. Since biosynthesis is comprised of other metabolic pathways besides protein synthesis, equations (11–13) are just approximations. They are good approximations because the cell dry weight is to a great extent composed of proteins. Whenever we make use of equations (11–13) we will write the sign ~ to indicate order of magnitude rather than equality.

More generally, the cell biomass is composed of proteins, lipids, ribonucleotides, nucleotides, and sugars. Therefore, instead of considering proteome fractions it is better to define mass fractions relative to the cell dry weight. In this generalized context *b*, *σ* and *β* are effective parameters. *ρbλ* is the biomass fraction of the biosynthetic machinery. *σ* is the energy required to duplicate the cell biomass per unit of dry weight. *β* is the number of carbon atoms required to duplicate the cell biomass per unit of dry weight. Finally, the pre-factor *ρ* in equations (8–10) takes into account that we express metabolic rates per cell volume instead of per cell mass.

Equation (8) is only valid when the right hand side is greater than zero. *ϕ*_*0*_ + *bλ* is the biomass fraction occupied by non-metabolic biomass components plus the biosynthetic machinery (mostly ribosomes). When *ϕ*_*0*_ + *bλ* = 1, all the biomass is represented by the non-metabolic biomass and the biosynthetic machinery and there is no biomass fraction left for energy generating pathways. Therefore, growth can be sustained only for *ϕ*_*0*_ + *bλ* < 1, defining the maximum growth rate


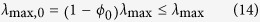


where


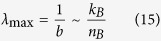


*λ*_max_ is the maximum growth rate determined by the autocatalytic nature of the biosynthetic machinery: ribosomes synthesize the ribosomes, which is proportional to the ribosome efficiency (*k*_*B*_) and inversely proportional to the number of amino acids in a ribosome (*n*_*B*_). In turn, *λ*_max,0_ is the correction to *λ*_max_ taking into account that a fraction of the biomass accounts for non-metabolic components. As expected, adding non-metabolic components reduces the maximum feasible growth rate (14).

Basan *et al*.^11^ make the additional assumption that *ϕ*_*0*_ is independent of the growth rate, which is essential to obtain a mixed OxPhos/fermentation in their model. However, while the proteome can indeed be divided into those four fractions, their subdivision in itself does not impose any constraint on the growth dependence (or independence) of *ϕ*_*0*_. Indeed, in the calculations presented above we have made no assumption about the growth dependence, or independence, of the biomass fraction associated with non-metabolic components *ϕ*_*0*_. In the following we analyse the consequences of accounting for a variable *ϕ*_*0*_.

### First scenario: *ϕ*_*0*_ is variable, but molecular crowding is neglected

**Figure 1:**
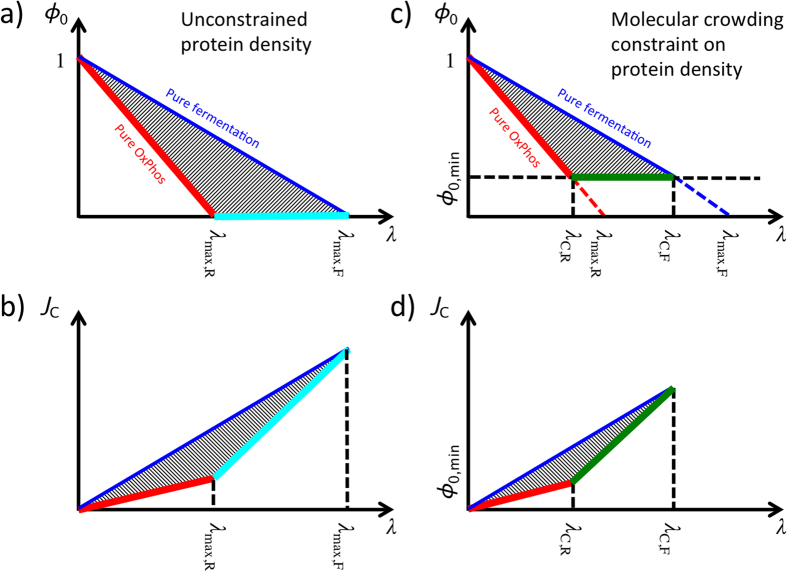
Solution space of the simplified model of Basan *et al*.^11^. The red line represents the solution with pure OxPhos. The blue line represents the solution with pure fermentation. The dashed area represents solutions with mixed OxPhos/fermentation. The thick lines correspond with the optimal solution minimizing carbon uptake as a function of the proliferation rate, which is discussed in the main text. There are no feasible solutions in the white background outside the triangle. (**a**,**b**) Solution space when neglecting molecular crowding. The cyan line represents the optimal solution with mixed OxPhos/fermentation. (**c**,**d**) Solution space when accounting for molecular crowding. The green line represents the optimal solution with mixed OxPhos/fermentation. *λ*_max,*R*_ and *λ*_max,*F*_ are the maximum growth rate determined by the autocatalytic nature of the biosynthetic machinery taking into account the energy cost of biosynthesis, when the energy demand is satisfied by pure OxPhos and fermentation, respectively. λ_*C,R*_ and λ_*C,F*_ are the corrections to *λ*_max,*R*_ and *λ*_max,*F*_, respectively, when there is a biomass fraction of non-metabolic proteins.

In this case *ϕ*_*0*_ becomes an additional variable and there is no unique solution to equations (8–10). The full solution space as a function of (*λ*, *ϕ*_*0*_) can be obtained by analysing the extreme cases of energy generation by OxPhos or fermentation alone and taking into account that 0 < *λ* < ∞ and 0 < *ϕ*_*0*_ < 1 (Fig. 1a,b). Since the biomass associated with *ϕ*_*0*_ does not contribute to metabolism, it contributes to carbon consumption without any benefit for growth. Therefore, the optimal solution minimizing carbon uptake coincides with the solution minimizing *ϕ*_*0*_. In the following we focus on the optimal solution that maximizes the growth rate given a carbon uptake rate *J*_*C*_. The optimal solution is to satisfy the energy demand with the pathway with higher ATP yield per carbon atom consumed (highest *e*_*i*_). For *E. coli e*_*F*_ = 2 and *e*_*R*_ = 4.4^11^ and, as expected, the optimal solution is to satisfy the energy demand with OxPhos. More precisely


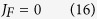



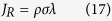



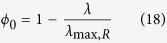


This solution is valid for 0 < *λ* < *λ*_max,R_. If *ε*_*R*_ < *ε*_*F*_ (as observed ref. 11) then there are feasible solutions for *λ* > *λ*_max,R_. For *λ* > *λ*_max,R_ the optimal solution that maximizes the growth rate given a carbon uptake rate *J*_*C*_ is *ϕ*_*0*_ = 0 and, consequently, *ρ*_*0*_ = 0, *i.e.*, a zero density of non-metabolic components. In this range of growth rates we obtain a mixed fermentation/OxPhos solution. To be more explicit, the full optimal solution is


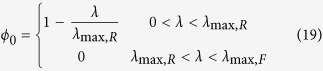



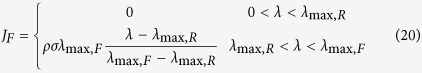



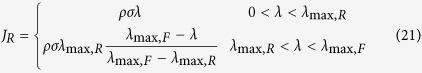


where


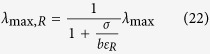



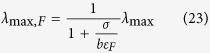


*λ*_max,R_ and *λ*_max,F_ are the maximum growth rate determined by the autocatalytic nature of the biosynthetic machinery taking into account the energy cost of biosynthesis, when the energy demand is satisfied by pure OxPhos and fermentation, respectively. There is no feasible solution for *λ* > *λ*_max,F_.

The biological interpretation of equations (19–21) is as follows. Starting for a zero growth rate, as the growth rate increases there is an increase in the biomass fraction allocated to metabolism (biosynthesis and energy generation), with a concomitant decrease of the non-metabolic biomass fraction. In this regime, the biomass fraction associated with metabolism is allowed to expand and, therefore, we say it is unconstrained. However, once the biomass fraction allocated to metabolism occupies the whole biomass, and, therefore, we say it is constrained. In the mixed OxPhos (*λ*_max,R_ < *λ* < *λ*_max,F_) the metabolic biomass has reached its maximum capacity (the whole biomass) and feasible solutions can only take place by using metabolic pathways with a lower requirement of biomass fraction.

The solution obtained above has some disagreements with experimental observations. First, the model predicted growth rate threshold for overflow metabolism is about five fold above the observed growth rate threshold for overflow metabolism. Using the parameter estimates reported by Basan *et al*.^11^ we obtain *λ*_max,R_ ≈ 4.2/h while the observed threshold is 0.78/h^11^. Second, in the mixed OxPhos/fermentation phase the model predicts *ϕ*_*0*_ = 0, while the actual observed value is *ϕ*_*0*_ ≈ 0.8^11^. Thus, there is discrepancy in the model derived and experimentally observed results.

### Second scenario: *ϕ*_*0*_ is variable but molecular crowding is accounted for

Biological and physical considerations exclude the possibility of *ϕ*_*0*_ = 0. The biomass fraction of non-metabolic components can be calculated as *ϕ*_*0*_ = *ρ*_*0*_*/ρ*, where *ρ*_*0*_ is the biomass density of non-metabolic components and *ρ* is the biomass density. Thus how small *ϕ*_*0*_ can be is dependent on how small *ρ*_*0*_ and how large *ρ* can be. Regarding *ρ*_*0*_, cells for example need a cytoskeletal support to maintain their size and shape. The biomass associated with the cytoskeleton should be proportional to the cell volume and, consequently, its density (mass per unit of cell volume) must be nonzero or, more precisely, larger than a minimum value *ρ*_*0*,min_. Regarding *ρ*, molecular crowding imposes a maximum macromolecular density *ρ*_max_.

The maximum macromolecular density together with a non-zero density of non-metabolic biomass components implies a minimum biomass fraction of non-metabolic components:


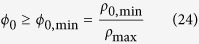


This lower limit to *ϕ*_*0*_ reduces the space of feasible solutions (Fig. 1c,d). As in the previous case, the optimal solution that maximizes the growth rate given a carbon uptake rate *J*_*C*_ is the one with minimum *ϕ*_*0*_, resulting in


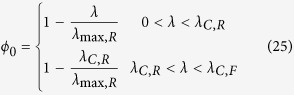



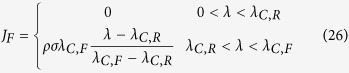



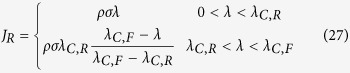


where


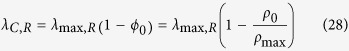



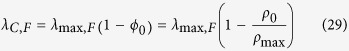


are the corrections to *λ*_max,R_ and *λ*_max,F_, respectively, when there is a biomass fraction of non-metabolic components. We note that (Eq. 25) predicts a growth independent *ϕ*_*0*_ in the range *λ*_*C*,R_ < *λ* < *λ*_*C*,F_. Not surprisingly, for *λ*_*C*,R_ < *λ* < *λ*_*C*,F_ the expressions for the fermentation and OxPhos rates (equations (26) and (27)) are equivalent to those obtained by Basan *et al*.^11^ assuming that *ϕ*_*0*_ is fixed.

The biological interpretation of equations (25–28) is similar to the previous case, with one correction. Starting for a zero growth rate, as the growth rate increases there is an increase in the biomass fraction allocated to metabolism (biosynthesis and energy generation), with a concomitant decrease of the non-metabolic biomass fraction. In this regime, the biomass fraction associated with metabolism is allowed to expand and, therefore, we say it is unconstrained. However, once the biomass fraction allocated to metabolism occupies the maximum allowed value (1 − *ϕ*_*0*,min_), there is no cytoplasmic space for further increase and, therefore, we say it is constrained. In the mixed OxPhos (*λ*_*C*,R_ < *λ* < *λ*_*C*,F_) the metabolic biomass has reached its maximum capacity (1 − *ϕ*_*0*,min_) and feasible solutions can only take place by using metabolic pathways with a lower requirement of biomass fraction. In this case the threshold for the switch is *λ*_*C*,R_. Based on the parameter estimates reported by Basan *et al*.^11^ *λ*_*C*,R_ is about 0.8/h, close to the experimentally observed value of 0.78/h.

Since *ϕ*_0_ is predicted to be growth dependent for 0 < *λ* < *λ*_*C*,R_ and *ϕ*_*0*_ = *ρ*_*0*_*/ρ*, our calculations imply that *ρ*_*0*_, *ρ* or both are growth dependent for 0 < *λ* < *λ*_*C*,R_ as well. In one possible scenario, *ρ*_*0*_ could be growth independent and *ρ* increase with increasing the growth rate. We have previously observed that the cell buoyant density of *E. coli* cells increases when increasing the growth rate from 0.1 to 0.4 volume exchange per hour^4^, and remains approximately constant above 0.4/hr. This observation supports an increase of the biomass density *ρ* in the growth rate range preceding overflow metabolism. In another possible scenario, *ρ* could be growth independent and *ρ*_*0*_ decrease with increasing the growth rate until the minimum value *ρ*_*0*,min_ is reached. A decrease in *ρ*_*0*_ could be achieved by relaxing the definition of non-metabolic biomass to include the fraction of metabolic enzymes that are effectively inactive. For example, some fraction of ribosomes may be disassembled or inactive, effectively contributing to the biomass fraction that is not actively catalysing metabolic reactions. Thus, with increasing the growth rate, an increase of the fraction of metabolic enzymes that are actually catalysing their corresponding reactions can be effectively represented by a growth dependent decrease of the density of non-metabolic biomass. In either case, the essence of the metabolic switch from OxPhos to mixed OxPhos/fermentation is a switch from unconstrained metabolic biomass (it can increase at expenses of reducing *ϕ*_*0*_) to a constrained (fixed to the maximum allowed value) metabolic biomass; and the maximum metabolic biomass is determined the ratio between the minimum possible density of non-metabolic biomass and the maximum macromolecular density.

The analysis presented here does not provide any evidence in favour or in contradiction with these two scenarios. The key conclusion of our analysis is that, because of molecular crowding, the decrease in *ϕ*_*0*_ with increasing the growth rate cannot continue beyond a threshold growth rate.

## Discussion

We obtained the full solution of the model of *E. coli* metabolism recently developed by Basan *et al*.^11^ as a function of the growth rate and the biomass fraction of non-metabolic proteins *ϕ*_*0*_ (Fig. 1). We note that, for any given growth rate, a lower *ϕ*_*0*_ indicates a lower rate of the carbon uptake required to sustain growth. Therefore, as the growth rate increases, a metabolic switch from pure OxPhos (Fig. 1a, red line) to mixed OxPhos/fermentation with *ϕ*_*0*_ = 0 (Fig. 1a, cyan line) is the optimal solution. If this solution were observed experimentally, then protein allocation alone could explain overflow metabolism. Yet, since experimental data indicate that *ϕ*_*0*_ > 0^11^, any satisfactory theory of overflow metabolism must incorporate additional factors to explain why the solution with *ϕ*_*0*_ = 0 is not observed in nature.

*ϕ*_*0*_ is, by definition, the ratio between the density of non-metabolic macromolecules and all macromolecules. If we assume that cells need a minimum macromolecular density to satisfy non-metabolic functions *ρ*_*0*,min_, then only a very high intracellular macromolecular density achieves *ϕ*_*0*_ = 0. However, molecular crowding imposes an upper limit on the maximum macromolecular density *ρ*_max_. When we account for this upper limit, the optimal solution is a metabolic switch from pure OxPhos (Fig. 1c, red line) to mixed OxPhos/fermentation with 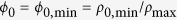 (Fig. 1c, green line). Importantly, even though we do not impose any constraint on *ϕ*_*0*_, our model with molecular crowding predicts that it is greater than zero and growth independent in the mixed OxPhos/fermentation regime. Therefore, the solution obtained by Basan *et al*.^11^ is in fact just a particular instantiation of our more general theory presented here.

That said, the experimental data reported by Basan *et al*.^11^ provides the first direct experimental estimate of the fermentation and OxPhos rates per unit of proteome in *E. coli*. Their data shows that fermentation generates about twice as much ATP per unit of fermentation proteome than OxPhos per unit of OxPhos proteome. Translated to the molecular crowding description, OxPhos requires about 2 times more space than fermentation to generate the same amount of energy.

We note that the cytoplasmic space associated with a given pathway includes both the pathway enzymes localized to the cytoplasm and the ribosomes required to synthesize those enzymes. For example, even though the OxPhos machinery localizes to the plasma membrane in bacteria, the biosynthesis of OxPhos enzymes requires some ribosomes in the cytoplasm. Further analysis will be required to discriminate between the molecular crowding of the plasma membrane by OxPhos enzymes^12^ and the molecular crowding of the cytoplasm by the associated biosynthetic pathways.

In eukaryotes the OxPhos enzymes are found in mitochondria that are embedded in the cytoplasm. Therefore, OxPhos has a dual contribution to molecular crowding, by its mitochondrial localization in the cytoplasm and by the required biosynthetic machinery. The component due to localization to the cytoplasm is independent of growth, indicating the possibility of overflow metabolism in non-growing, high-energy-need cells^9^. We note that in mammalian cells, OxPhos requires between 5 to 50 times more space than fermentation to produce the same amount of energy^7^, establishing macromolecular crowding as the cause of overflow metabolism from bacteria to mammalian cells.

One could postulate an upper limit or optimal macromolecular density without making any reference to molecular crowding. However, a more complete theory of overflow metabolism is needed to explain why macromolecules occupy about 30–40% of cell volume^13^. A hard limit (that cannot be exceeded) is imposed by the finite sizes and shapes of macromolecules. For example, polydisperse spheres can be packed to a maximum volume fraction of about 80%^14^. A lower, “soft” limit (that can be exceeded, but becomes suboptimal) is imposed by the fact that molecular crowding slows down diffusion. The competition between the tendency to increase reaction rate by increasing the concentration of enzymes, and the decrease in diffusion rate when increasing the macromolecular fraction predicts the existence of an optimal macromolecular fraction^15^. For *E. coli*, the predicted optimal macromolecular fraction is approximately 37%^15^. This number agrees well with the measured macromolecular volume fraction range for *E. coli*, 34–44%^13^. To our knowledge there is no other current theory that achieves such quantitative agreement.

We conclude that the metabolic switch from OxPhos to mixed OxPhos/fermentation (overflow metabolism) is a switch from unconstrained to constrained metabolic biomass. More generally, a satisfactory explanation of the switch from high- to low-yield metabolism with an increasing metabolic rate requires three major components: (i) a higher pathway rate per unit of pathway biomass for the low-yield pathway, (ii) a non-zero density of non-metabolic macromolecules, and (iii) an upper bound in the cell macromolecular density. Since molecular crowding explains (iii), molecular crowding is a key factor in explaining overflow metabolism.

## Additional Information

**How to cite this article**: Vazquez, A. and Oltvai, Z.N. Macromolecular crowding explains overflow metabolism in cells. *Sci. Rep.* **6**, 31007; doi: 10.1038/srep31007 (2016).
